# Combined effects of strength training, whey protein, and HMB on sarcopenia in older people: A systematic review and meta-analysis

**DOI:** 10.1097/MD.0000000000050477

**Published:** 2026-09-04

**Authors:** Jordan Hernandez-Martinez, Izham Cid-Calfucura, Edgar Vásquez-Carrasco, Braulio Henrique Magnani Branco, Pedro Delgado-Floody, Santos Villafaina, Carlos Cristi-Montero, Tomás Herrera-Valenzuela, Pablo Valdés-Badilla

**Affiliations:** a Department of Physical Activity Sciences, Universidad de Los Lagos, Osorno, Chile; b Department of Education, Faculty of Humanities, Universidad de La Serena, La Serena, Chile; c Department of Physical Activity, Sports and Health Sciences, Faculty of Medical Sciences, Universidad de Santiago de Chile (USACH), Santiago, Chile; d School of Occupational Therapy, Faculty of Psychology, Universidad de Talca, Talca, Chile; e Centro de Investigación en Ciencias Cognitivas, Faculty of Psychology, Universidad de Talca, Talca, Chile; f Graduate Program in Health Promotion, Cesumar University (UniCesumar), Maringá, Brazil; g Department of Physical Education, Sport, and Recreation, Universidad de La Frontera, Temuco, Chile; h Didáctica de la Expresión Musical, Plástica y Corporal, Facultad de Ciencias del Deporte, Grupo de Investigación en Actividad Física, Calidad de Vida y Salud (AFYCAV), Universidad de Extremadura, Avenida de la Universidad s/n, Cáceres, España; i Instituto de Investigación e Innovación en Deporte (INIDE), Cáceres, España; j IRyS Group, Physical Education School, Pontificia Universidad Católica de Valparaíso, Valparaíso, Chile; k Department of Physical Activity Sciences, Faculty of Education Sciences, Universidad Católica del Maule, Talca, Chile; l Sports Coach Career, Faculty of Life Sciences, Universidad Viña del Mar, Viña del Mar, Chile.

**Keywords:** energy intake, healthy aging, muscle strength, nutrition therapy, resistance training

## Abstract

**Background:**

Although previous studies have reported the benefits of combining physical activity with supplementation on body composition and physical performance in older people with sarcopenia, previous meta-analysis often included heterogeneous interventions, combining multiple types of supplements. Therefore, this systematic review and meta-analysis aimed to analyze the available body of published peer-reviewed randomized controlled trials (RCTs) studies on the effects of strength training (ST) combined with supplementation (S) (ST + S) using whey protein (WP) and beta-hidroxi-beta-metilbutirato (HMB) on body composition, physical performance, and biomarkers in older people with sarcopenia.

**Methods:**

Using 6 databases PubMed, Medline, Cumulative Index to Nursing and Allied Health Literature Complete, Scopus, Cochrane Library, and Web of Science a thorough literature search was carried out to March 2025. The Grading of Recommendations Assessment, Development, and Evaluation, Tool for the assEssment of Study qualiTy and reporting in EXercise, Rob 2, and Preferred Reporting Items for Systematic reviews and Meta-Analyses tools were used to evaluate the methodological quality and certainty of evidence. Hedge *g* effect sizes were computed. Potential sources of heterogeneity, such as subgroup analyses (type of supplements or training vs supplementation), were chosen using a fixed- or random-effects model. The protocol (code CRD42025643858) was registered in International Prospective Register of Systematic Reviews.

**Results:**

Of 2322 records, 7 RCTs with 472 participants were included. These 7 studies showed a high methodological quality ≥10 points in Tool for the assEssment of Study qualiTy and reporting in EXercise. Four overall meta-analysis showed significant increases (*P *< .05) in fat-free mass (effect sizes [ES] = 0.39), maximal isometric handgrip strength (MIHS, ES = 1.38), chair stand test (ES = 0.33), and 6-minute walk test (ES = 0.33). In the subgroup meta-analysis, the ST + S subgroup showed a significant increase (*P* < .01) in MIHS compared to supplementation alone. Similarly, WP combined with ST was associated with a significant improvement in MIHS (ES = 0.49), whereas no significant improvement was observed with HMB combined with ST. In contrast, in fat mass and knee extension strength, no significant differences were reported between ST + S and ST or S.

**Conclusion:**

ST combined with WP or HMB supplementation may contribute to improvements in fat-free mass and selected physical performance outcomes in older people with sarcopenia. However, the limited number of RCTs, relatively small sample sizes, moderate-to-high risk of bias, and inconsistent subgroup findings indicate that the current evidence should be interpreted cautiously and considered promising rather than definitive.

## 1. Introduction

Sarcopenia is a geriatric syndrome that consists of a decrease in skeletal muscle mass and a decline in functional physical capacity.^[1]^ It affects between 10% and 16% of older people worldwide.^[2]^ Sarcopenia is related to adverse health outcomes, such as disability, impaired quality of life, increased risk of hospitalization, and mortality.^[3]^ Therefore, it is essential to implement interventions aimed at improving these altered indicators through physical activity, nutritional strategies, or a combination of both.^[4]^ However, strength training (ST) is one of the most widely used types of physical activity intervention to treat sarcopenia,^[5]^ with the most commonly used methods being resistance training (employing free weights and progressive loading machines) and elastic band training with exercises for both the upper and lower body.^[5–7]^

In a meta-analysis focused on older people with sarcopenia,^[5]^ significant improvements in maximal isometric handgrip strength (MIHS, *P *< .001), gait speed (*P *< .001), and skeletal muscle index (*P *< .001) were reported in favor of resistance training using free weights compared with active/inactive control groups (CG). Similarly, a meta-analysis in older people with sarcopenia,^[8]^ showed significant improvements in MIHS (*P *< .001) and skeletal muscle index (*P *< .001) in favor of elastic band training over active/inactive CG. However, in a meta-analysis in older people with sarcopenia,^[7]^ there were no significant improvements in MIHS (*P *= .57), but there were significant improvements in timed up-and-Go (TUG, *P *< .001), gait speed (*P *< .001), and chair stand test (CST, *P *< .001) in favor of ST with varied load machines compared to active/inactive CG.

Regarding supplementation as a treatment, there is evidence of interventions with supplementation alone and supplementation combined with physical activity.^[9]^ In a meta-analysis focused on older people with sarcopenia,^[9]^ significant improvements in appendicular muscle mass (*P *= .04) were found only with whey protein (WP), leucine, vitamin D supplementation, and combined aerobic and muscle strength exercise (*P *= .01). However, significant improvements in MIHS (*P *< .001) and short physical performance battery (*P *< .001) were observed only in the group that performed physical activity combined with supplementation. Similarly, a meta-analysis in older people with sarcopenia^[10]^ reported significant increases in skeletal muscle mass index (*P *< .05) and appendicular skeletal muscle mass (*P *< .05) in favor of the WP supplementation group compared to the isocaloric placebo group. In terms of physical performance, only improvements in MIHS (*P *< .01) were reported in favor of WP supplementation combined with ST versus isocaloric placebo.

Although previous studies have reported the benefits of combining physical activity with supplementation on body composition and physical performance in older people with sarcopenia,^[9,10]^ previous meta-analyses often included heterogeneous interventions, combining multiple types of supplements. This limits the ability to discern the specific contribution of individual compounds such as whey protein or beta-hydroxy-beta-methyl-butyrate (HMB). Therefore, there is a need for a focused synthesis of evidence that isolates the effects of these specific supplements when combined with ST, in order to inform more targeted clinical and nutritional strategies in the management of sarcopenia.^[11–13]^ In this regard, the present meta-analysis aimed to analyze the available set of published peer-reviewed randomized controlled trials (RCTs) on the effect of ST combined with WP or HMB on primary outcomes, such as body composition and physical performance. Secondly, on biomarkers in older people with sarcopenia. It is hypothesized that ST combined with WP or HMB leads to increases in fat-free mass (FFM), muscle strength, and cardiorespiratory fitness in older people with sarcopenia.

## 2. Methods

### 2.1. Protocol and registration

The Preferred Reporting Items for Systematic reviews and Meta-Analyses guidelines were adhered to in this systematic review.^[14]^ The protocol of this systematic review and meta-analysis has been registered in International Prospective Register of Systematic Reviews (ID code: CRD42024614097).

### 2.2. Eligibility criteria

The inclusion criteria for this systematic review and meta-analysis were met by original peer-reviewed articles published until March 2025 that were unrestricted by language or publication date. The materials excluded were conference abstracts, books, book chapters, editorials, letters to the editor, protocol records, reviews, case studies, and trials. In addition, this systematic review used the population, intervention, comparator, outcome, and study design framework (Table 1).

**Table 1:** Selection criteria used in the systematic review and meta-analysis.

Category	Inclusion criteria	Exclusion criteria
Population	People with (participants with sarcopenia). Participants were required to be elderly (age ≥ 60 yr-old), but there were no restrictions by sex or setting (such as hospitals, communities, or nursing homes).	Population under 60 yr of age only with sarcopenia and/or osteopenia. People over 60 yr-old apparently healthy.
Intervention	Interventions included resistance training, such as elastic resistance training and progressive resistance training, combined with supplementation with whey protein or hydroxymethylbutyrate.	Interventions that do not use strength training. There are no details of the intervention procedure.
Comparator	Interventions with or without an active/inactive CG or placebo.	Observational studies (i.e., cross-sectional, retrospective, and prospective studies) that do not include structured comparison pre/post analysis.
Outcome	Primary outcomes: body composition (e.g., fat-free mass, fat mass), or physical function (e.g., maximal isometric handgrip strength (MIHS), knee extention strength (KES), 30-s chair stand, 6-min walk test (6MWT)). Secondary outcome: biomarkers, and supplementation: whey protein and hydroxymethylbutyrate supplementation	Lack of baseline data and/or follow-ups.
Study design	Experimental design studies (randomized controlled trials) with pre- and post-assessments.	Non-randomized controlled trials, cross-sectional, retrospective, and prospective studies.

### 2.3. Information search process and databases

The search was conducted between October 2024 and March 2025 using 6 generic databases: PubMed, Medline, Cumulative Index to Nursing and Allied Health Literature Complete, Scopus, Cochrane Library, and Web of Science (core collection). The US National Library of Medicine Medical Subject Headings used free language terms related to ST, body composition, physical performance, and biomarkers in older people with sarcopenia. The search string used was as follows: (resistance training OR resistance exercise OR strength training) AND (“body composition” OR “body fat” OR “fat-free mass” OR “fat mass” OR “muscle mass” OR “body mass index” OR “nutritional status” OR “bone mineral density” OR “skeletal muscle mass index”) AND (“functional independence” OR “functional dependence” OR “functional mobility” OR “health condition” OR “falls” OR “fall risk” OR “risk of fall” OR “falling risk” “balance” OR “static balance” OR “dynamic balance” OR “walking speed” OR “gait speed” OR “mobility” OR “strength” OR “muscle strength” OR “upper body strength” OR “lower body strength” OR “muscle power”) AND (“biomarkers” OR “protein” OR “supplementation” OR “hydroxymethylbutyrate”) AND (“elderly” OR “older people” OR “older people” OR “older subject” OR “aging” OR “aging” OR “aged”) AND (“sarcopenic” OR “sarcopenia”). To assist in locating additional relevant studies, 2 independent experts were consulted regarding the included publications and the inclusion and exclusion criteria. We stipulated 2 requirements for the experts: to hold a PhD in sports science and to have peer-reviewed publications on physical activity in various population groups and/or physical performance published in journals with an impact factor according to the Journal Citation Reports. We did not disclose our search strategy to specialists to avoid bias in their searches. After completing these steps, we conducted the database search on March 10, 2025, for relevant retractions or errata related to the listed papers.

### 2.4. Studies selection and data collection process

The EndNote reference manager (version X9, Clarivate Analytics) exported the studies. JHM and ICC conducted separate searches, eliminated duplicates, examined titles and abstracts, and examined the complete texts. No disparities were observed at this time. The procedure was repeated for recommendations made by external research and searches of the reference lists. The texts of potentially suitable papers were then examined, and the rationale behind excluding those not fitting the selection criteria was disclosed.

### 2.5. Methodological quality assessment

Tool for the assEssment of Study qualiTy and reporting in EXercise (TESTEX), a tool for exercise-based intervention studies,^[15]^ assessed the methodological quality of the selected studies. One potential exclusion criterion was the TESTEX results,^[15]^ with a score of <8 points. According to Smart, Waldron, Ismail, Giallauria, Vigorito, Cornelissen, and Dieberg,^[15]^ there is a 15-point rating system (5 points for study quality and 10 points for reporting). Two authors (JHM and ICC) carried out this process separately, while a 3rd author (THV) served as a referee for cases that were borderline and needed further validation by another author (PVB).

### 2.6. Data synthesis

The following data were obtained and analyzed from the selected studies: author and year of publication; country of origin; study design; sex and mean age of the sample; number of participants in the intervention and control group; body composition assessment tool; diagnostic criteria for sarcopenia; type of supplement; training volume (total duration, weekly frequency, and time per session); training intensity; primary outcome; secondary outcome; adverse events; and adherence.

### 2.7. Risk of bias in individual studies

Two independent researchers (JHM and ICC) evaluated the risk of bias version 2 (RoB 2) of the included studies, and a 3rd researcher (PVB) analyzed the results. The Cochrane Handbook for Systematic Reviews of Interventions’ recommendations for RCTs was the foundation of this evaluation.^[16]^ Based on the randomization procedure, departures from the planned interventions, missing outcome data, outcome assessment, and choice of the reported result, the risk of bias was categorized as “high,” “low,” or “some concerns.”

### 2.8. Summary measures for meta-analysis

Meta-analyses were performed only when ≥3 studies were available.^[17]^ Effect sizes (ES; Hedge *g*) for each body composition and physical performance in the ST + supplementation (ST + S) and control group (CG) were calculated using the pretraining and post-training mean and standard deviation for each dependent variable. After calculating the magnitude of the difference between an experimental and CG using Hedges *g* test, the data were standardized using the change score (standard deviation). ES values are presented with 95% confidence intervals. Calculated ES was interpreted using the following scale: trivial, <0.2; small: 0.2 to 0.6; moderate: >0.6 to 1.2; large: >1.2 to 2.0; very large: >2.0 to 4.0; and extremely large, >4.0.^[18]^ Fixed- and random-effects models were used to account for differences between studies that might have affected the effect of ST. Comprehensive Meta-analysis software (version 2.0; Biostat) was used to perform these calculations. Statistical significance was set at *P* ≤ .05^[19]^ and was used to perform these calculations. In each trial, the random-effects model (Der Simonian–Laird approach) was used to calculate and pool the standard mean differences and mean differences of FFM, fat mass (FM), MIHS dominant and nondominant hand, knee extension strength (KES), CST, and 6-minute walk test (6MWT) strength training + supplementation vs CG (ST + S vs CG). The fundamental premise of the random-effects model is that genuine effects (interventions, duration, etc) vary across studies and that samples are selected from populations with varying effect sizes. Data were pooled if at least 3 studies showed the same results.^[20]^

Heterogeneity between trial results was tested using Cochran *Q* test and the *I*^2^ statistic. *I*^2^ values of <25%, 25% to 50%, and >50% represent small, medium, and large amounts of inconsistency, respectively.^[21]^ Egger regression tests were performed to detect small study effects and possible publication biases.^[22]^

### 2.9. Moderator analysis

Potential sources of heterogeneity likely to influence training effects were selected a priori using a random-effects model and an independent computed single-factor analysis. When low heterogeneity existed, a fixed model was used for the corresponding analysis.

### 2.10. Subgroup analysis

Given the adaptive responses to ST programs with supplementation,^[13,23]^ which may vary in response to training alone or supplementation-alone groups, as well as different types of supplements, these factors were considered possible moderating variables.

### 2.11. Certainty of evidence

Studies were categorized as having high, moderate, low, or very low confidence based on their assessment using the Grading of Recommendations Assessment, Development, and Evaluation scale.^[24]^ As studies with RCT designs were included, all analyses began with a high degree of certainty and were downgraded if there were concerns about bias, consistency, accuracy, precision, directness of results, or risk of publication bias.^[24]^ Two authors evaluated the studies separately (JHM and ICC), and any disagreements were settled by agreement with a 3rd author (EVC).

## 3. Results

**Figure 1.**
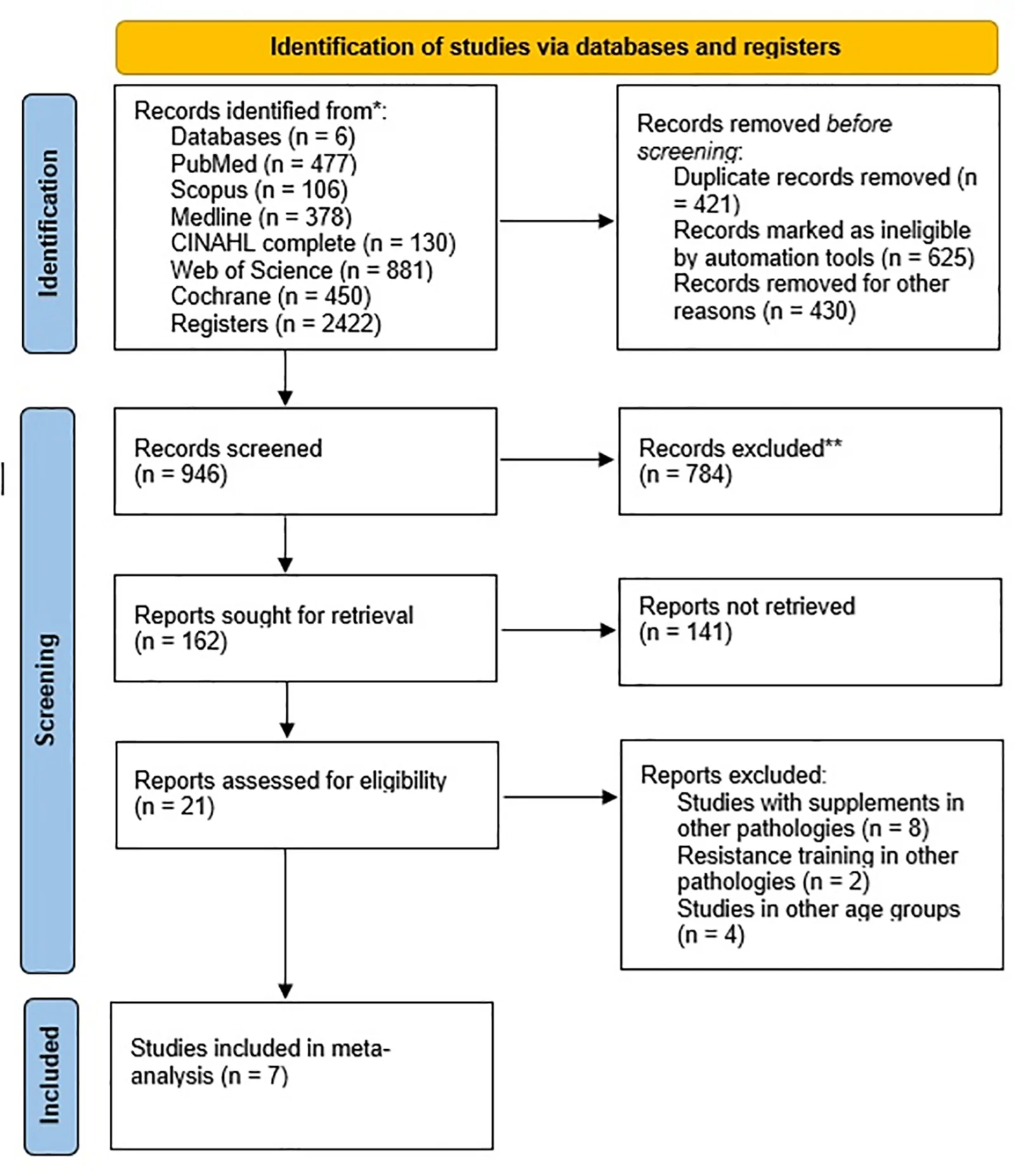
Flowchart of the review process. Based on PRISMA guidelines.^[14]^ PRISMA = Preferred Reporting Items for Systematic reviews and Meta-Analyses.

Figure 1 shows the search process used for the studies. A total of 2422 records were identified. Subsequently, duplicates were removed and the studies were filtered by selecting the title, abstract, and keywords, resulting in 946 references. In the subsequent analysis phase, 784 articles were excluded because they did not meet the search criteria, leaving 162 articles. Subsequently, 43 studies were descriptive, 21 were non-randomized controlled trials, 10 studies had more than 1 supplementation, 8 studies had ST, 12 studies had supplements, 32 sarcopenia associated with other pathologies, and 15 were systematic reviews. Reports were assessed for eligibility, 21 of which 15 were excluded from 8 studies with supplements in other pathologies, 2 ST in other pathologies, and 4 studies in other age groups. Seven studies remain to be meta-analyzed.^[25–31]^

### 3.1. Methodological quality

Seven selected studies were analyzed using the TESTEX scale (Table 2). All these studies achieved a score equal to or >60% on the scale,^[25–31]^ namely 10/15,^[30]^ 11/15,^[26]^ 12/15,^[25]^ 13/15,^[28,29]^ and 14/15.^[27]^

**Table 2:** Study quality assessment according to the TESTEX scale.

Elegibility criteria specified	Randomly allocated participants	Allocation concealed	Gorups similar at baseline	Assessors blinded	Outcome measures assesed > 85% of participants*	Intention to treat analysis	Reporting of between group statistical comparisons	Point measures and measures of variability reported†	Activity monitoring in control group	Relative exercise intensity reviewed	Exercise volume and energy expended	Overall TESTEX‡
Amasene, Cadenas-Sanchez^[25]^	Yes	Yes	Yes	Yes	Yes (1)	No	Yes	Yes (2)	Yes	No	Yes	12/15
Han, Ji^[26]^	Yes	No	Yes	No	Yes (1)	No	Yes	Yes (2)	Yes	Yes	Yes	11/15
Liao, Liao^[27]^	Yes	Yes	Yes	Yes	Yes (1)	Yes	Yes	Yes (2)	Yes	Yes	Yes	14/15
Meza-Valderrama, Sánchez-Rodríguez^[28]^	Yes	Yes	Yes	Yes	Yes (1)	Yes	Yes	Yes (2)	Yes	No	Yes	13/15
Mori and Tokuda^[29]^	Yes	Yes	Yes	Yes	Yes (1)	No	Yes	Yes (2)	Yes	Yes	Yes	13/15
Shahar, Kamaruddin^[30]^	Yes	No	Yes	No	Yes (1)	No	Yes	Yes (2)	Yes	No	Yes	10/15
Zhu, Chan^[31]^	Yes	Yes	Yes	Yes	Yes (1)	Yes	Yes	Yes (2)	Yes	No	Yes	13/15

TESTEX = Tool for the assEssment of Study qualiTy and reporting in EXercise.

* Three points are possible: 1 point if adherence > 85%, 1 point if adverse events were reported, and 1 point if exercise attendance was reported.

† Two points possible: 1 point if the primary outcome is reported, 1 point if all other outcomes were reported.

‡ Total out of 15 points. TESTEX: tool for assessing study quality and reporting in exercise.

### 3.2. Risk of bias

**Figure 2.**
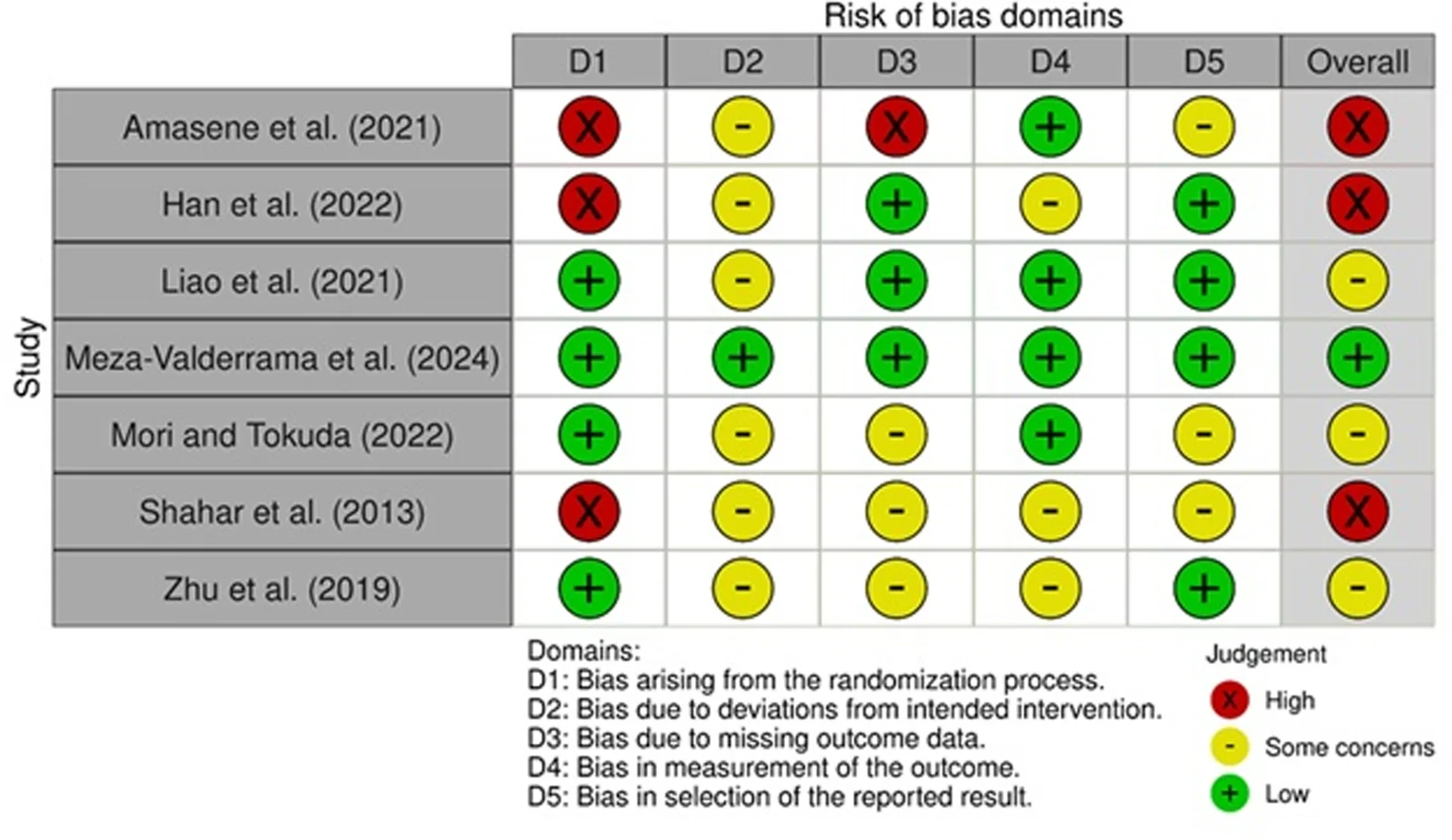
Risk of bias within studies. D1, randomization process; D2, deviations from the intended interventions; D3, missing outcome data; D4, measurement of the outcome; D5, selection of the reported results.

**Figure 3.**
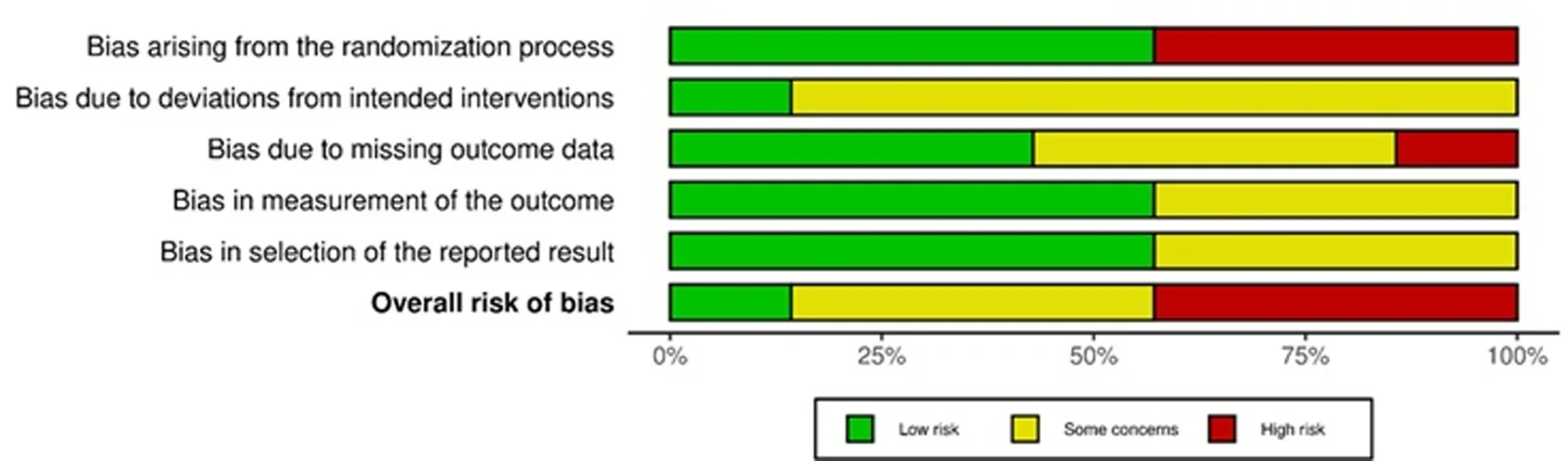
Risk of bias summary: review the authors and judgments about each risk of bias item in each included study.

Figures 2 and 3 show the risk of bias assessments. One study was assessed as having a low risk of bias across all domains.^[28]^ Three studies showed concerns in 1 or more domains.^[27,29,31]^ Three studies were classified as having a high risk of bias.^[25,26,30]^ Overall, the risk of bias was moderate to high, with most studies presenting some concerns and only a few demonstrating low risk across all domains.

### 3.3. Studies and sample characteristics

All studies were RCTs, of which 5 were conducted in Asia,^[26,27,29–31]^ and 2 in Europe.^[25,28]^ Of these studies, 5 presented both male and female in the sample, ≥60% being female,^[26,28–31]^ while only 2 studies presented only female in the sample,^[25,27]^ with an mean age of 75.4 ± 5.63 years, with a total of 472 participants (>70% female), 191 in the experimental group and 281 in the CG. Of the experimental groups, 5 used supplementation with WP + ST,^[25,27,29–31]^ and 2 were supplemented with HMB + ST.^[26,28]^ In all ST interventions, upper- and lower-body exercises were performed using resistance training^[25,26,28–30]^ and elastic band training.^[27,31]^ The duration of the interventions ranged from 12 weeks with frequencies of 2 to 3 sessions/wk for 40 to 60 minutes,^[25–31]^ with intensities between 50% and 80% of the 1RM,^[26,29]^ and 13 to 15 points on the Borg scale.^[27]^ All studies showed primary outcomes and 2 presented secondary outcomes.^[25,26]^ No adverse events were reported in any study, and adherence was ≥80% in all the studies. The results are presented in detail in Table 3.

**Table 3:** Strength training with or without supplementation and its effects on body composition and physical performance in older people with sarcopenia.

Authors	Study design	Country	Sex (age)	Groups (Sample size)	Body composition assessment tool	Diagnostic criteria for sarcopenia	Type of supplement	Training dosage and intensity									Primary outcome		Secondary outcome	Adverse events	Adherence
								Weeks	Frequence			Minutes				Intensity	Body composition	Physical performance and functional capacity in activities of daily living	Biomarkers		
Amasene, Cadenas-Sanchez^[25]^	RCT	Spain	Women (100%) ST + S: 82.9 ± 5.67 ST + P: 81.2 ± 6.14	ST + S (21): resistance training + whey protein ST + P (20): resistance training + placebo	DXA	(EWGSOP2)	Protein	12	2			60				NR	ASM (kg) Fat-free mass (kg) -at mass (kg) Calf circumference (cm)	SPPB total score (pts) Frail (N, %) MIHS (kg)	Myostatin (ng/mL) Follistatin (ng/mL) Follistatin to myostatin ratio Irisin (µg/mL)	NR	NR
Han, Ji^[26]^	RCT	China	75% women 25% men ST + S: 78.3 ± 5.9 ST: 78.5 ± 6.5 S: 77.1 ± 6.5	ST + S (43): resistance training + HMB ST (45): resistance training S (44): HMB	DXA	(AWGS)	(HMB)	12	3				60		70%–80% 1RM		Fat mass (kg) SMI (kg/m^2^)	MIHS (kg) BI (pts)	NR	NR	NR
Liao, Liao^[27]^	RCT	China	Women 100% ST + S: 68.6 ± 7.42 ST: 69.8 ± 7.24	ST + S (36): elastic band training + whey protein ST (36): elastic band training	DXA	(EWGSOP2 and AWGS)	Protein	12		2				60	13 to 15 Borg RPE scale		SMI (kg/m^2^) AMI (kg/m^2^)	Walking speed (m/s)	NR	NR	>80%
Meza-Valderrama, Sánchez-Rodríguez^[28]^	RCT	Spain	Women 75% Men 25% ST + S: 81.8 ± 8.8 ST + P: 81.3 ± 10.2	ST + S (17): resistance training + HMB ST + P (15): resistance training + placebo	DXA	(EWGSOP2)	(HMB)	12		3	60				NR		Fat mass (kg) Fat-free mass (kg)	SPPB (pts) MIHS (kg) GS (m/s) BI (pts)	NR	NR	NR
Mori and Tokuda^[29]^	RCT	Japan	Women 85% Men 15% ST + S: 77.7 ± 3.3 ST: 77.6 ± 5.2 S: 77.8 ± 4.5	ST + S (23): resistance training + whey protein ST (23): resistance training S (24): whey protein	DXA	AWGS	Protein	12		3	40				50%–70% 1RM		AMI (kg/m^2^)	MIHS (kg) KES (kg) Walking speed (m/s)	NR	NR	NR
Shahar, Kamaruddin^[30]^	RCT	Malaysia	Women 75% Men 25% ST + S: 65.20 ± 4.87 ST: 69.74 ± 5.46 S: 65.93 ± 4.37	ST + S (15): resistance training + whey protein ST (19): resistance training S (15): whey protein	DXA	(EWGSOP2)	Protein	12		2	60				NR		Fat-free mass (kg) Body fat (%)	MIHS (kg) TUG (sec) 6MWT (m) Chair Stand Test (rep)	Protein carbonyl (nmol/L) Superoxide dismutase (specific activity) Lipid hydroperoxide (mmol/L) IL-6 (pg/mL) IGF-1 (pg/mL) Orexin (ng/mL)	NR	NR
Zhu, Chan^[31]^	RCT	Japan	Women 60% Men 40% ST + S: 74.8 ± 6.9 ST: 74.5 ± 7.1	ST + S (36): elastic band training + whey protein ST (40): elastic band training	DXA	AWGS	Protein	12		2	60				40% 1RM		ASM (kg)	MIHS (kg) KES (kg) 6MWT (m) Chair Stand Test (rep)	NR	NR	

% = percentages, 1RM = 1-repetition maximum, 6WMT = 6-walk minutes test, AWGS = Asian Working Group for Sarcopenia, DXA = dual X-ray absorptiometry, EWGSOP2 = European Working Group on Sarcopenia in Older People, HMB = hydroxymethylbutyrate, IGF-1, KES = knee extension strength, MIHS = maximal isometric handgrip strength, NR = not reported, RCT = randomized controlled trials, rep = repetitions, S = supplementation, ST = strength training, ST + S = strength training + supplementation.

### 3.4. Meta-analysis results

#### 3.4.1. Primary outcome

The overall effects of ST combined with WP and HMB supplementation on these variables are shown in Table 4. The forest plots are shown in Figures S1–S6, Supplemental Digital Content 1. There were significant moderate and large effects (*P* < .05) in favor of ST for FFM and MIHS (ES = 0.39–1.38). In the FM, KES, and CST, there were no significant differences (*P* > .05) with small to moderate effect sizes (ES = 0.07–0.37).

**Table 4:** Effects of strength training combined with whey protein and hydroxymethylbutyrate supplementation on body composition, physical performance and biomarkers in older people with sarcopenia.

	n	Model of effect	ES (95% CI)	*P*	*I^2^* (%)	Egger test (*P*)	RW (%)
Body composition							
Fat-free mass (kg)	3, 3, 4, 109	Fixed	0.39 (0.04–0.74)	**.02**	**0.00**	**.87**	5.17–12.2
Fat mass (kg)	3, 3, 4, 192	Fixed	0.07 (−0.18 to 0.32)	.57	**0.00**	**.95**	0.64–4.34
Physical performance							
MIHS (kg)	6, 6, 9, 400	Random	1.38 (0.10–2.65)	**.001**	63.9	.00	19.8–20.1
KES (kg)	2, 2, 3, 146	Fixed	0.26 (−0.12 to 0.65)	.17	**38.6**	**.19**	3.89–6.54
Chair stand test (rep/s)	2, 2, 3, 125	Fixed	0.33 (0.009–0.66)	**.04**	**25.1**	**.26**	5.25–8.51
6MWT (m)	2, 2, 3, 125	Fixed	0.33 (0.008–0.66)	**.04**	**37.0**	**.20**	4.87–7.36

The results marked in bold are significant.

6MWT = 6-minute walk test, CI = confidence interval, ES = effect sizes, KES = knee extension strength, MIHS = maximal isometric handgrip strength, rep = repetions, RW = relative weight.

#### 3.4.2. Meta-analysis subgroup

##### 3.4.2.1. Subgroup analysis by type supplementation

In MIHS, there were significant differences in ST + S versus S (*P* = .002) and not significant differences in ST + S versus ST (*P* = .06) in ST versus S (*P* = .06). Regarding the type of supplementation, significant differences were reported in favor of ST + WP CG (ES = 0.49) and not in ST + HMB versus CG (ES = 0.55) in MIHS. These results are presented in Figures S7 and S8, Supplemental Digital Content 2.

#### 3.4.3. Secondary outcome

Regarding biomarkers, it was impossible to perform meta-analysis because of the small number of studies.^[25,30]^ When reported qualitatively, there were no significant improvements in favor of ST + S concerning CG (*P* ≥ .05) in the biomarkers myostatin (ng/mL), follistatin (ng/mL), and follistatin/myostatin irisin ratio (g/mL).^[25]^ On the other hand, in a study,^[30]^ significant improvements in the levels of protein carbonyl (nmol/L) were reported in favor of ST + S compared to CG (*P* < .05). In addition, significant improvements in the levels of lipid hydroperoxide (mmol/L) were identified in the ST + S group compared with control condition. No significant improvements were reported in favor of ST + S in the biomarkers of superoxide dismutase, interleukin-6 (pg/mL), insulin-like growth factor (pg/mL), and orexin (ng/mL) compared to a control condition.

### 3.5. Certainty of evidence

The existing evidence is insufficient to make definitive recommendations regarding ST + S interventions aimed at improving body composition and physical performance in older people with sarcopenia. Although some studies have reported encouraging outcomes, these findings emphasize the need for additional research to establish more conclusive results and create evidence-based strategies tailored to this population (Table 5).

**Table 5:** GRADE assessment for the certainty of evidence.

Assessment of certainty							Number of patients		Effect		Certainty	Importance
Number of studies	Study design	Risk of bias	Inconsistency	Indirect evidence	Vagueness	Other considerations	Strength training with or without supplementation	Active control group	Relative (95% CI)	Absolute (95% CI)		
Effects of resistance training intervention along with leucine-enriched whey protein supplementation on sarcopenia and frailty in post-hospitalized older people: preliminary findings of a randomized controlled trial												
1	Randomized trials	Very serious	It’s not serious	It’s not serious	It’s not serious	None	21/41 (51.2%)	20/41 (48.8%)	Not estimable		⨁⨁ ◯◯ Go down	Important
Effect of resistance training combined with βhydroxy-β-methylbutyric acid supplements in elderly patients with sarcopenia after hip replacement												
1	Randomized trials	Very serious	It is not serious	It is not serious	It is not serious	None	132/177 (74.6%)	45/177 (25.4%)	Not estimable		⨁⨁ ◯◯ Go down	Important
Effects of protein-rich nutritional composition supplementation on sarcopenia indices and physical activity during resistance exercise training in older women with knee osteoarthritis												
1	Randomized trials	Serious†	It is not serious	It is not serious	It is not serious	None	36/72 (50.0%)	36/72 (50.0%)	Not estimable		⨁⨁⨁ ◯ Moderate†	Important
Supplementation with β-hydroxy-β-methyl-butyrate after resistance training in post-acute care patients with sarcopenia: A randomized, double-blind placebo-controlled trial												
1	Randomized trials	It is not serious	It is not serious	It is not serious	It is not serious	None	17/32 (53.1%)	15/32 (46.9%)	Not estimable		⨁⨁⨁⨁ High	Important
De-training effects following leucine-enriched whey protein supplementation and resistance training in older people with sarcopenia: a randomized controlled trial with 24 wk of follow-up												
1	Randomized trials	Serious†	It is not serious	It is not serious	It is not serious	None	46/70 (65.7%)	24/70 (34.3%)	Not estimable		⨁⨁⨁ ◯ Moderate†	Important
Effectiveness of exercise and protein supplementation intervention on body composition, functional fitness, and oxidative stress among elderly Malays with sarcopenia												
1	Randomized trials	Very serious	Serious†	It is not serious	It is not serious	None	49/65 (75.4%)	16/65 (24.6%)	Not estimable		⨁ ◯◯◯ Very low*^,^†	Not important
Effects of exercise and nutrition supplementation in community-dwelling older Chinese people with sarcopenia: a randomized controlled trial												
1	Randomized trials	Serious†	It is not serious	It is not serious	It is not serious	None	76/113 (67.3%)	37/113 (32.7%)	Not estimable		⨁⨁⨁ ◯ Moderate†	Important

CI = confidence interval, GRADE = Grading of Recommendations Assessment, Development, and Evaluation.

* High.

† Some concerns.

## 4. Discussion

This meta-analysis aimed to analyze the effect of ST combined with WP or HMB on the primary outcomes, namely body composition and physical performance, and secondarily on biomarkers in older people with sarcopenia. Our meta-analysis reported significant improvements in FFM (*P* = .02; ES = 0.39), MIHS in both hands (*P* = .001; ES = 1.38), CST (*P* = .04; ES = 0.33), and 6MWT (*P* = .04; ES = 0.33) in favor of the ST + supplementation groups concerning CG. Conversely, our subgroup analysis by type of supplementation reported significant differences in ST + S versus S for MIHS (*P* = .002; ES = 0.63). Regarding the type of supplementation, significant differences were reported in favor of ST + WP versus CG (*P* = .02; ES = 0.49) and ST + HMB versus CG (*P* = .04; ES = 0.55) in MIHS.

### 4.1. Primary outcome

#### 4.1.1. Body composition

##### 4.1.1.1. Fat-free mass

Significant improvements were observed in FFM (*P* = .002; ES = 0.39) in favor of the ST + supplementation groups compared with the CG. This finding is consistent with a previous meta-analysis evaluating WP supplementation combined with ST in young and older people, which also reported significant improvements in FFM in favor of the ST + S group compared with placebo (*P* < .001).^[32]^ However, these findings should be interpreted cautiously given the limited number of studies available. Our meta-analysis included 3 studies with a total of 4 experimental groups. Two studies combined ST with WP supplementation,^[25,30]^ whereas only 1 study prescribed HMB supplementation.^[28]^ When the studies were examined individually, no significant improvements in FFM were observed. However, when the data (n, mean, and standard deviation) were pooled pre- and post-intervention for the experimental and CG, a significant overall effect in favor of ST combined with supplementation was identified. This discrepancy may reflect the increased statistical power provided by meta-analysis and the limited sample sizes of the individual trials, rather than consistent effects across all studies. The observed improvements are biologically plausible. Previous evidence suggests that WP supplementation combined with ST may enhance muscle protein anabolism,^[33,34]^ whereas HMB supplementation has been proposed to attenuate muscle mass loss by promoting protein synthesis through mechanisms involving the PI3K/Akt-dependent mTOR signaling pathway.^[35–38]^ Nevertheless, because the present meta-analysis was not designed to evaluate the underlying mechanisms and included a limited number of heterogeneous studies, these physiological explanations should be considered speculative.

##### 4.1.1.2. Fat mass

No significant improvements were observed in FM (*P* = .57; ES = 0.07) in favor of the ST + S group compared with the CG. This finding is consistent with previous meta-analyses evaluating WP supplementation combined with ST in young and older people, which likewise reported no significant improvements in FM compared with placebo or control groups.^[10,32]^ The absence of a significant effect should be interpreted cautiously given the limited number of studies included in this analysis. Notably, 2 of the 3 studies contributing to this outcome did not report the training intensity,^[25]^ a factor that may influence changes in body composition following ST interventions in older people.^[39]^ In addition, differences in participant characteristics may have contributed to the observed findings. For example, 1 study included a high proportion of participants with severe sarcopenia and functional impairment following recent hospitalization due to an acute illness.^[28]^ Consequently, the response of FM to ST combined with supplementation may vary according to the severity of sarcopenia and the methods used to assess body composition, which may have contributed to the variability observed across studies.^[37]^

#### 4.1.2. Physical performance

##### 4.1.2.1. Maximal isometric handgrip strength

Significant improvements in MIHS in both hands (*P* = .001; ES = 1.38) were found in favor of the supplemented ST groups compared with CG. This is similar to that reported in a meta-analysis with WP,^[9]^ leucine, and vitamin D supplementation interventions in patients with sarcopenia who reported significant improvements in MIHS when a physical activity program was used in combination with nutritional supplementation (*P* = .0009). However, when physical activity was not combined with WP, leucine, or vitamin D supplementation, no significant improvements in MIHS were reported in older people with sarcopenia. This is in line with the results of a meta-analysis with ST interventions in older people with secondary sarcopenia^[8]^ who identified significant improvements in MIHS in favor of ST interventions (*P* < .01) compared to CG. However, this is in contrast to the findings of a meta-analysis of machine-based progressive ST interventions in older people with sarcopenia,^[7]^ who reported no significant improvements in MIHS (*P* = .57). In contrast, a recent meta-analysis^[10]^ incorporating RCT of WP supplementation with and without ST in older people with sarcopenia reported significant increases in MIHS (*P* < .05) in the protein-supplemented and ST groups compared to a placebo group that only performed their daily routine. Although our findings support the potential benefits of combining ST with nutritional supplementation to improve MIHS, they should be interpreted cautiously because the available evidence is based on a limited number of RCTs. Collectively, the available evidence suggests that ST appears to be an important component of interventions targeting MIHS in older people with sarcopenia, whereas supplementation may provide additional benefits in some individuals.^[5]^ The observed improvements may be related to the neuromuscular demands of resistance exercise involving the upper limbs, which has been associated with adaptations in muscle strength and muscle mass following repeated training.^[40,41]^ However, the present meta-analysis was not designed to identify the physiological mechanisms responsible for these adaptations.

##### 4.1.2.2. Knee extension strength

No significant improvements were observed for KES (*P* = .17; ES = 0.26) in favor of the ST plus supplementation groups compared with the CG. To our knowledge, this is the 1st meta-analysis to examine KES in older people with sarcopenia. However, these findings should be interpreted cautiously because the analysis was based on only 2 studies comprising 3 intervention groups. Although the pooled analysis did not demonstrate a significant overall effect, the individual studies reported heterogeneous findings. One RCT reported significant improvements in KES following ST combined with WP supplementation compared with WP supplementation alone (*P* < .01).^[29]^ In addition, the group receiving WP supplementation alone showed a smaller improvement in KES than the groups performing ST with or without supplementation.^[29]^ Similarly, another study reported significant improvements in both the ST plus supplementation group and the ST-alone group compared with the CG.^[31]^ Taken together, these findings suggest that ST may play an important role in improving lower-body muscle strength in older people with sarcopenia, whereas the additional contribution of nutritional supplementation remains uncertain. The absence of a significant pooled effect may reflect the limited number of available studies, small sample sizes, and methodological differences between interventions rather than the absence of a true effect. Although ST has been associated with neuromuscular adaptations and improvements in muscle strength,^[40,42,43]^ the present meta-analysis was not designed to evaluate these mechanisms. Therefore, further well-designed RCTs are needed to clarify the effects of combining ST with nutritional supplementation on KES.

##### 4.1.2.3. Chair stand test

Significant improvements were observed in the CST (*P* = .04; ES = 0.33) in favor of the ST plus supplementation groups compared with the CG. This finding is consistent with a previous meta-analysis of machine-based progressive ST interventions in older people with sarcopenia, which also reported significant improvements in CST compared with the CG (*P* < .001).^[7]^ In contrast, a more recent meta-analysis evaluating WP supplementation with and without ST found no significant improvements in CST (*P* = .94) in the protein-supplemented groups.^[10]^

The differences between these findings may reflect variations in study characteristics, including intervention duration, participant characteristics, sample size, and the type of supplementation administered. Therefore, the present findings should be interpreted cautiously, particularly considering the limited number of available studies. Previous evidence suggests that ST performed 2 to 3 times per week can improve muscle strength in older people,^[5,42,43]^ and this training frequency was generally consistent with the interventions included in the present review. Likewise, previous meta-analytic evidence has shown that elastic bands training performed for 40 to 60 minutes/session, at least 3 times/wk for 12 weeks, may improve muscle strength in older people with sarcopenia.^[5]^ Although nutritional supplementation may complement the adaptive response to ST in some individuals,^[10]^ the present findings do not allow conclusions regarding its independent contribution to CST performance.

##### 4.1.2.4. 6-minute walk test

Significant improvements were observed in the 6MWT (*P* = .04; ES = 0.33) in favor of the ST plus supplementation groups compared with the CG. However, this finding should be interpreted cautiously because it was based on only 2 studies. Both interventions implemented 12 weeks of ST using elastic bands that included lower-body exercises, whereas the experimental groups also received WP supplementation.^[30]^ One possible explanation for the observed improvement is that repeated lower-body resistance exercise enhanced muscle strength and force-generating capacity, thereby facilitating walking performance in older people.^[44]^ It is also plausible that nutritional supplementation contributed to preserving or increasing muscle mass and supporting recovery, which may have complemented the adaptations induced by ST. Nevertheless, these mechanisms were not directly evaluated in the included studies and therefore should be considered speculative.

### 4.2. Subgroup analysis by type of supplementation in MIHS

The subgroup analysis should be interpreted cautiously because it was based on indirect comparisons and a limited number of studies. Significant improvements in MIHS were observed in the ST + WP subgroup compared with the CG (*P* = .02), whereas the ST + HMB subgroup did not reach statistical significance (*P* = .054). These findings should not be interpreted as evidence that WP is superior to HMB, as the available data were derived from different studies with distinct participant characteristics and intervention protocols.

WP is considered a high-quality protein source because of its high biological availability and rapid digestion compared with other dietary proteins. Previous evidence suggests that protein intakes of approximately 1.5 to 1.6 g/kg/d may maximize muscle protein synthesis in older people, although the optimal intake for individuals with sarcopenia remains uncertain.^[45]^ In addition, ST has consistently been shown to stimulate muscle protein synthesis, improve muscle strength, and attenuate the progression of sarcopenia.^[5]^ It is therefore plausible that combining ST with WP may enhance these adaptations by supporting muscle protein accretion and recovery through pathways such as mTOR.^[9,42]^ Although these mechanisms are biologically plausible, they were not evaluated directly in the studies included in this review and should, therefore, be interpreted with caution.

For HMB supplementation, no statistically significant effect was observed, although the pooled estimate approached significance (*P* = .054). HMB has been proposed to influence skeletal muscle metabolism by stimulating protein synthesis through mTOR signaling and increasing circulating IGF-1 concentrations.^[46]^ Likewise, several studies have suggested that combining HMB supplementation with ST may improve muscle mass and muscle strength.^[47,48]^ Nevertheless, an overview of systematic reviews concluded that the effects of HMB supplementation on muscle strength remain inconsistent or inconclusive.^[37]^ The limited number of studies evaluating HMB combined with ST in older people with sarcopenia, together with differences in participant characteristics, intervention protocols, and diagnostic criteria for sarcopenia or frailty, may partly explain these inconsistent findings.^[37]^

### 4.3. Subgroup analysis according to the combination of strength training and supplementation for MIHS

Significant differences were observed between ST + S and S alone (*P* = .002) for MIHS. This finding is consistent with previous evidence indicating that combining ST with nutritional S may enhance improvements in muscle strength compared with S alone.^[10]^ In contrast, S without ST has generally shown limited effects on muscle strength, although favorable changes in some morphological outcomes have been reported in older people with sarcopenia.^[9]^ One possible explanation is that ST induces neuromuscular adaptations, including improvements in motor unit recruitment and firing frequency,^[30]^ whereas S alone is unlikely to elicit these training-specific adaptations.

No significant differences were observed between ST + S and ST (*P* = .06) or between ST and S (*P* = .06). These findings should be interpreted cautiously because they were derived from a limited number of studies and subgroup analyses with relatively small sample sizes. Although the absence of a significant difference between ST + S and ST may suggest that the additional effect of S on MIHS is modest, it does not exclude the possibility of clinically meaningful benefits in specific populations or for other outcomes, such as FFM.^[32]^ Likewise, the lack of a significant difference between ST and S contrasts with the overall pattern reported in the literature and may reflect limited statistical power and methodological differences across the included studies rather than equivalent effects of the interventions.

### 4.4. Secondary outcome

Regarding biomarkers, only a limited number of studies evaluated these outcomes, precluding quantitative synthesis. One study^[25]^ reported no significant differences between ST + S and the CG for myostatin, follistatin, or the follistatin/myostatin ratio (all *P* ≥ .05). These findings differ from those of another study,^[49]^ which reported an 18% increase in follistatin following elastic band ST in older women, although both studies were consistent in showing no significant changes in myostatin. Previous studies evaluating myostatin responses to ST have also reported inconsistent findings, with both increases and decreases observed following training interventions.^[50–52]^ Similarly, no significant changes in irisin were observed, in agreement with previous findings in apparently healthy older people following resistance and strength-endurance training.^[53]^ Given the limited number of available studies and their methodological heterogeneity, further research is needed to clarify the effects of ST combined with nutritional S on these biomarkers.

In contrast, another study^[30]^ reported significant reductions in protein carbonyl and lipid hydroperoxide concentrations following ST + S compared with the control condition (*P* < .05), suggesting a potential reduction in oxidative stress. Although these findings are encouraging, they should be interpreted cautiously because they are based on a single study. However, no significant between-group differences were observed for SOD or IGF-1. Despite the decrease in SOD observed in the ST group compared with the ST + S group, the clinical significance of this finding remains unclear. Likewise, the absence of changes in IGF-1 contrasts with previous findings from a 16-week ST intervention in middle-aged and older women that reported increased circulating IGF-1 concentrations.^[54]^ Differences in intervention duration, weekly training frequency, and participant characteristics may partly explain these discrepant findings. Finally, IL-6 concentrations remained unchanged following the interventions. Previous evidence suggests that changes in IL-6 may depend on factors such as exercise modality and training intensity,^[54–56]^ which could also have contributed to the variability observed across studies.

Overall, the available evidence regarding biomarker responses remains limited, as only a small number of studies evaluated these outcomes, and substantial methodological heterogeneity precluded quantitative synthesis. Consequently, no firm conclusions can be drawn regarding the biological mechanisms underlying the improvements in muscle strength and physical function observed in the present review. Further well-designed RCTs incorporating standardized biomarker assessments are needed to clarify the biological mechanisms associated with ST combined with nutritional S in older people with sarcopenia.

### 4.5. Clinical implications

Clinical heterogeneity should also be considered when interpreting the present findings. Although the pooled analyses provide an overall estimate of the effects of ST combined with nutritional S, the included studies differed in several clinically relevant aspects, including participant characteristics, baseline nutritional status, diagnostic criteria for sarcopenia, ST prescription (exercise modality, intensity, and duration), S protocols (type and dosage), intervention supervision, and adherence. These methodological and clinical differences may have contributed to the variability of the pooled estimates and should be considered when interpreting the overall findings. Consequently, the present findings should be generalized with caution, and future RCTs should adopt more standardized intervention protocols, outcome measures, and diagnostic criteria to improve the comparability of findings.

From a clinical perspective, the present findings reinforce the role of ST as the cornerstone intervention for the management of sarcopenia. Although nutritional S combined with ST was associated with improvements in several clinically relevant outcomes, subgroup analyses did not consistently demonstrate additional benefits over ST alone, particularly for MIHS. These findings suggest that nutritional S should currently be considered a complementary strategy that may enhance the response to appropriately prescribed ST in some individuals, rather than a replacement for or a clearly superior intervention to ST alone. Given the limited number of available studies and the clinical heterogeneity among interventions, further adequately powered RCTs are required to identify which individuals are most likely to benefit from the addition of nutritional S and to optimize individualized treatment strategies in clinical practice.

### 4.6. Practical applications

Based on the available evidence, individualized ST programs may be considered for older people with sarcopenia, with exercises targeting large muscle groups, intensities ranging from approximately 50% to 80% of 1-repetition maximum or a Borg rating of perceived exertion of 13 to 15, and a training volume of 2 to 3 sessions/wk lasting 40 to 60 minutes. Whenever feasible, supervision by a qualified professional may facilitate proper exercise execution and improve adherence, particularly during the initial stages of the intervention.^[57]^ Nutritional S should currently be considered a complementary strategy to ST rather than a replacement for exercise. The available evidence suggests that WP supplementation (20–40 g/d) may be considered, particularly in individuals with inadequate protein intake or increased protein requirements, whereas HMB supplementation (typically 3 g/d) may represent an additional option in selected individuals. However, the optimal S strategy remains uncertain, and decisions regarding the type and dosage of S should be individualized according to the patient’s nutritional status, clinical characteristics, and preferences. Regular assessment of body composition, muscle strength, and functional capacity may help clinicians monitor treatment response and guide individualized adjustments to exercise and nutritional interventions. Nevertheless, additional well-designed RCTs with larger sample sizes are needed to establish the optimal exercise prescription, determine the additional contribution of nutritional S, and identify the patient populations most likely to benefit from these combined interventions. Future studies should also incorporate standardized biomarker assessments to better understand the biological mechanisms underlying the observed clinical improvements.

## 5. Limitations and strengths

Our meta-analysis presented certain limitations: the number of studies considered in the meta-analysis was small, which could affect the accuracy of our results; the risk of bias was moderate to high, with most studies raising some concerns and only a few demonstrating a low risk; differences in the diagnostic criteria used to identify sarcopenia across the included studies may have contributed to variability in participant characteristics and treatment responses. Heterogeneity may also have been influenced by differences in ST protocols, including the use of machines, free weights, and elastic bands, as well as variations in training intensity and exercise selection. Furthermore, differences in protein dosage and the baseline nutritional status of participants may have modified the response to S. These factors should be considered when interpreting the pooled findings and may partially explain the observed between-study heterogeneity; and the intervention time in all studies was short (12 weeks), which underlines the need for future research with a longer duration to validate our results. On the other hand, the strengths of our meta-analysis include: a methodological quality >60% in the analyzed studies; the use of methodological processes governed by Preferred Reporting Items for Systematic reviews and Meta-Analyses, International Prospective Register of Systematic Reviews, TESTEX, RoB 2, and Grading of Recommendations Assessment, Development, and Evaluation; the use of 6 generic databases: PubMed, Medline, Cumulative Index to Nursing and Allied Health Literature Complete, Scopus, and Web of Science; the performance of a meta-analysis by subgroup according to the type of S; and the comparison by subgroups according to the combination of ST with and without S.

## 6. Conclusion

ST combined with nutritional S (WP or HMB) may improve FFM, MIHS, CST performance, and 6MWT distance in older people with sarcopenia. However, these findings should be interpreted cautiously because they are based on only 7 RCTs with relatively small sample sizes and an overall moderate-to-high risk of bias. Furthermore, subgroup analyses did not consistently demonstrate additional benefits of combining nutritional S with ST compared with ST alone, particularly for MIHS. Therefore, nutritional S should currently be considered a complementary strategy to appropriately prescribed ST rather than a replacement for, or a clearly superior alternative to, exercise alone. The available evidence should be considered promising rather than definitive. Further well-designed RCTs with larger sample sizes, longer intervention periods, adequately powered comparisons, and standardized biomarker assessments are needed to clarify the additional contribution of nutritional S, as well as the biological mechanisms underlying the observed functional improvements, before firm clinical recommendations can be made.

## Acknowledgments

The authors thank the editorial board and the anonymous peer reviewers for their constructive feedback during manuscript revision.

## Author contributions

**Conceptualization:** Jordan Hernandez-Martinez, Pablo Valdés-Badilla.

**Data curation:** Jordan Hernandez-Martinez, Pablo Valdés-Badilla.

**Formal analysis:** Jordan Hernandez-Martinez, Izham Cid-Calfucura, Edgar Vásquez-Carrasco, Pablo Valdés-Badilla.

**Investigation:** Jordan Hernandez-Martinez, Izham Cid-Calfucura, Edgar Vásquez-Carrasco, Braulio Henrique Magnani Branco, Pedro Delgado-Floody, Santos Villafaina, Carlos Cristi-Montero, Tomás Herrera-Valenzuela, Pablo Valdés-Badilla.

**Methodology:** Jordan Hernandez-Martinez, Edgar Vásquez-Carrasco, Pablo Valdés-Badilla.

**Resources:** Jordan Hernandez-Martinez.

**Software:** Jordan Hernandez-Martinez, Izham Cid-Calfucura, Pablo Valdés-Badilla.

**Supervision:** Jordan Hernandez-Martinez, Pablo Valdés-Badilla.

**Validation:** Jordan Hernandez-Martinez, Edgar Vásquez-Carrasco, Pablo Valdés-Badilla.

**Writing – original draft:** Jordan Hernandez-Martinez, Izham Cid-Calfucura, Edgar Vásquez-Carrasco, Pablo Valdés-Badilla.

**Writing – review & editing:** Jordan Hernandez-Martinez, Izham Cid-Calfucura, Edgar Vásquez-Carrasco, Braulio Henrique Magnani Branco, Pedro Delgado-Floody, Santos Villafaina, Carlos Cristi-Montero, Tomás Herrera-Valenzuela, Pablo Valdés-Badilla.
















